# New genomic resources and comparative analyses reveal differences in floral gene expression in selfing and outcrossing *Collinsia* sister species

**DOI:** 10.1093/g3journal/jkab177

**Published:** 2021-05-20

**Authors:** Lauren J Frazee, Joanna Rifkin, Dinusha C Maheepala, Alannie-Grace Grant, Stephen Wright, Susan Kalisz, Amy Litt, Rachel Spigler

**Affiliations:** 1 Department of Biology, Temple University, Philadelphia, PA 19122, USA; 2 Department of Ecology and Evolutionary Biology, University of Toronto, Toronto, ON M5S, Canada; 3 Department of Botany and Plant Sciences, University of California, Riverside, Riverside, CA 92521, USA; 4 Department of Ecology and Evolutionary Biology, University of Tennessee, Knoxville, TN 37996, USA

**Keywords:** *Collinsia*, RNA-seq, selfing syndrome, pollen, floral development, differential gene expression, DESeq2, dichogamy, evolutionary genomics, Hi-C scaffolding, parallel evolution

## Abstract

The evolutionary transition from outcross- to self-fertilization is one of the most common in angiosperms and is often associated with a parallel shift in floral morphological and developmental traits, such as reduced flower size and pollen to ovule ratios, known as the “selfing syndrome.” How these convergent phenotypes arise, the extent to which they are shaped by selection, and the nature of their underlying genetic basis are unsettled questions in evolutionary biology. The genus *Collinsia* (Plantaginaceae) includes seven independent transitions from outcrossing or mixed mating to high selfing rates accompanied by selfing syndrome traits. Accordingly, *Collinsia* represents an ideal system for investigating this parallelism, but requires genomic resource development. We present a high quality *de novo* genome assembly for the highly selfing species *Collinsia rattanii*. To begin addressing the basis of selfing syndrome developmental shifts, we evaluate and contrast patterns of gene expression from floral transcriptomes across three stages of bud development for *C. rattanii* and its outcrossing sister species *Collinsia linearis*. Relative to *C. linearis*, total gene expression is less variable among individuals and bud stages in *C. rattanii*. In addition, there is a common pattern among differentially expressed genes: lower expression levels that are more constant across bud development in *C. rattanii* relative to *C. linearis*. Transcriptional regulation of enzymes involved in pollen formation specifically in early bud development may influence floral traits that distinguish selfing and outcrossing *Collinsia* species through pleiotropic functions. Future work will include additional *Collinsia* outcrossing-selfing species pairs to identify genomic signatures of parallel evolution.

## Introduction

Parallel evolutionary phenomena give us exceptional opportunities to learn how adaptations arise and the nature of their underlying genetic basis. In plants, mating system change from outcrossing to selfing has occurred numerous, independent times throughout evolutionary history (Barrett 2002), and is one of the most common transitions across the angiosperms (Stebbins 1974). Moreover, self-fertilizing (hereafter selfing) species frequently converge on a constellation of traits known as the “selfing syndrome” (Lloyd 1979; Sicard and Lenard 2011). Selfing species commonly exhibit reduced flower size, pollen-to-ovule ratios, pollinator rewards, and floral longevity compared to their outcrossing ancestors (Eckert *et al.* 2009; Goodwillie *et al.* 2010; Kalisz *et al.* 2012). The repeated evolution of the selfing syndrome, combined with evidence of its potential ecological drivers (Kalisz *et al.* 2004; Fishman and Willis 2008; Pettengill and Moeller 2012; Toräng *et al.* 2017; Mazer *et al.* 2020), suggest that these complex phenotypes represent adaptations. However, our understanding of their molecular genetic basis and the extent to which genomic pathways to the syndrome are constrained or possible is minimal (Sicard and Lenhard 2011; Shimizu and Tsuchimatsu 2015; Blount *et al.* 2018; Tsuchimatsu *et al.* 2020; Woźniak *et al*. 2020).

**Figure 1:**
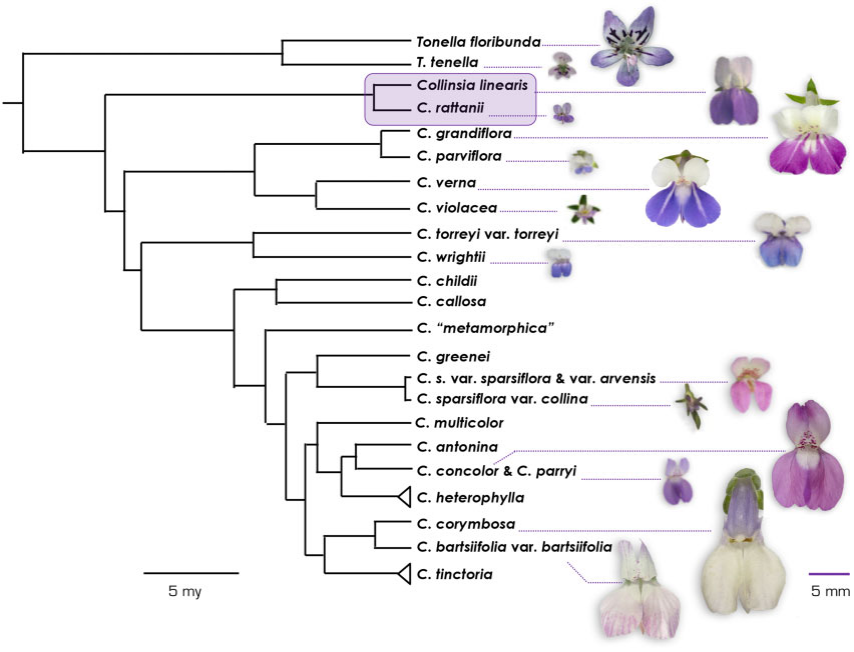
Phylogeny of *Collinsia* and sister taxa *Tonella* based on Bayesian phylogenetic analysis. Figure adapted from Baldwin *et al.* (2011). Branch lengths are scaled to time, in million years (black scale bar). Maximal basal calibration is at 15 Ma. Pictures of *Tonella* and *Collinsia* flowers belonging to sister species illustrating the repeated transition from large-flowered outcrossing species to small-flowered selfing species within both genera are shown. Pictures are to scale (purple scale bar). Focal species *C. rattanii* and its sister species *C. linearis* are highlighted in purple. Photos by Thomas Harper.

The genus *Collinsia* (Plantaginaceae) is an exemplary system for exploring the genomic basis of selfing syndrome traits and the mechanisms driving their parallel evolution. A phylogeny of *Collinsia* shows at least seven independent transitions from predominantly outcrossing to predominantly selfing, with outcrossing and selfing species falling into discrete groups based on flower size (Armbruster *et al.* 2002; Baldwin *et al.* 2011; Kalisz *et al.* 2012) (Figure 1). All *Collinsia* species are self-compatible in contrast to many current plant systems used to study selfing syndrome evolution (*e.g.*, *Arabidopsis*, *Capsella*, *Brassica*, and *Leavenworthia*, all Brassicaceae; *Solanum*, Solanaceae; and *Ipomoea* spp., Convolvulaceae: Vekemans *et al.* 2014; Rifkin *et al.* 2019; Durand *et al.* 2020; Tsuchimatsu *et al.* 2020), all of which exhibit a transition from self-incompatible, obligate outcrossing to highly selfing involving mutations at specific S-loci (but also see *Erythranthe*, formerly *Mimulus*: Wu *et al.* 2008). Consequently, the loss of self-incompatibility is not involved in the evolution of selfing in *Collinsia* and must have arisen, at least in part, through different developmental and molecular mechanisms. High rates of selfing in *Collinsia* species are linked to decreased temporal and spatial separation (dichogamy and herkogamy, respectively) of male and female phases within hermaphroditic flowers (Kalisz *et al.* 2012). Specifically, onset of stigmatic receptivity and stigma-anther contact occur significantly earlier in the floral lifespan of selfing *Collinsia* species than in their outcrossing sister species (Kalisz *et al.* 2012). Functionally, this means that self-pollen is deposited on stigmas of selfing species before opportunities to receive outcross pollen, and because selfing species have shorter floral lifespans, outcrossing opportunities are limited overall (Kalisz *et al.* 2012). In contrast, predominantly outcrossing *Collinsia* species self-fertilize only when sufficient outcross pollen fails to reach the stigma and/or ovules (*i.e.*, pollinator failure: Elle and Carney 2003; Kalisz and Vogler 2003; Kalisz *et al.* 2004). The abundance of ecological and phenotypic data on *Collinsia* species, including evidence of differences in range size and niche breadth between selfers and outcrossers (Randle *et al.* 2009; Grant and Kalisz 2020) and evidence of distinct ecological drivers of selfing across species pairs (*e.g.*, Kalisz *et al.* 2004; Randle *et al*. 2018) facilitates hypothesis testing about selfing syndrome evolution.

Morphological and developmental differences between sister species are commonly rooted in spatial and/or temporal differences in gene expression (Carroll 2008; Stern and Orgogozo 2008). Recent studies in selfing *Capsella* have linked convergent evolution of reduced flower size to convergent developmental processes and gene expression changes (Fujikura *et al.* 2018; Wo**ź**niak *et al.*^2020^). To identify differences in gene expression that might underlie the shift to selfing in *Collinsia*, we compare gene expression during flower development in a striking example of recently diverged *Collinsia* sister species with distinct developmental and morphological traits.

Here, we first present a high quality *de novo* genome assembly and functional annotation for the highly selfing *Collinsia rattanii* to develop genomic resources for *Collinsia*. Then, we evaluate and contrast patterns of gene expression across floral bud development for *C. rattanii* and its predominantly outcrossing sister species *C. linearis*, using floral transcriptomes at three developmental stages. Based on these patterns, we ask: (1) Do genome-wide gene expression patterns differentiate species and stages of floral bud development? (2) Do we see less variation in overall gene expression among *C. rattanii* individuals, reflecting greater homozygosity as a highly selfing species, and across bud stages within *C. rattanii*, reflecting its shorter, more rapid period of floral development? (3) Are there consistent differences in gene expression across stages between the species, and do these differences tend to occur in early or late bud development? Finally, we analyze predicted functions of differentially expressed genes to identify those potentially involved in the evolution of the selfing syndrome in *C. rattanii*.

## Materials and methods

### Study species

The genus *Collinsia* currently encompasses 22 species of self-compatible, spring-flowering, annual plants (Baldwin *et al.* 2011). The native range of the genus spans temperate North America with its center of diversity in the California Floristic Province (Baldwin *et al.* 2011). *Collinsia* flowers are bilaterally symmetrical, and the four immature stamens and the stigma lie in the proximal part of a folded ventral petal, or keel. Upon anthesis, the four anthers dehisce one by one as each filament lengthens toward the distal part of the keel, while the style elongates toward the mature anther(s) and the stigma becomes receptive. In this way, post-anthesis flowers can be divided into four discrete stages, each defined by the number of dehisced anthers (Kalisz *et al.* 1999). The stage at which the first mature anther sheds pollen generally occurs before the stigma becomes receptive, but the absolute and relative timing of these events, as well as the timing of stigma-anther contact, differs among species. In outcrossing *Collinsia*, the timing of stigmatic receptivity and stigma-anther contact occurs relatively late in the floral lifespan, after most of the anthers have dehisced (Kalisz *et al.* 2012), which increases the odds of outcrossing but allows for selfing once outcrossing opportunities have passed (*i.e.*, “delayed selfing” *sensu*Lloyd 1979). In highly selfing *Collinsia* species, stigmatic receptivity and stigma-anther contact occur early in the floral lifespan, approximately when the first anther in the flower becomes mature (Kalisz *et al.* 2012). Functionally, this elimination of dichogamy and herkogamy in selfing *Collinsia* species allows for early autonomous selfing, prior to the possibility of—and even preempting—outcross pollen receipt (*sensu*Lloyd 1979).

The sister species *C. rattanii* (selfing) and *C. linearis* (outcrossing) diverged _**∼**_1.45 Mya (Baldwin *et al.* 2011) (Figure 1). Both species are found in open, dry coniferous forest habitats, but *C. rattanii* has a greater geographic range and niche breadth than *C. linearis*, consistent with the general pattern seen for selfers and outcrossers (Grossenbacher *et al.* 2015). The range of *C. rattanii* extends from northern California north into Washington, while the outcrossing *C. linearis* is limited to northern California and southern Oregon, where their ranges overlap (Grant and Kalisz 2020). Like most species in the genus, both are diploid (*n* = 7: Garber 1956, 1958). The sister species differ markedly in flower size and shape (Figure 1, Supplementary Figure S1), floral longevity (55.3 hours _**±**_ SE 4.7 in *C. rattanii vs* 96.2 hours _**±**_ SE 5.4 in *C. linearis*), timing of stigmatic receptivity *[*floral stage 1, defined by the number of dehisced anthers sensu Kalisz *et al.* (1999), in *C. rattanii vs* between floral stages 3 and 4 in *C. linearis*], anther-stigma contact *[*floral stage 1, *sensu*Kalisz *et al.* (1999), in *C. rattanii vs* floral stage **∼**3 in *C. linearis*] and outcrossing rate in the wild (*t_m_* = 0.12 in *C. rattanii vs* 0.57 in *C. linearis*) (Randle *et al.* 2009; Kalisz *et al.* 2012).

### *De novo* genome sequencing, assembly, and annotation

We planted seeds of *C. rattanii* collected from Trinity County, CA (40_**°**_29′19.78″N, 123_**°**_ 9′56.56″W) and raised the plants in the University of Tennessee, Knoxville (TN, USA) greenhouse. We collected young leaves and meristems from a single plant and sent the sample for high molecular weight DNA extraction and Shotgun paired-end Illumina and Chicago^TM^ sequencing to create a *de novo* draft genome assembly (Dovetail Genomics, Santa Cruz, CA, USA). Shotgun and Chicago libraries were sequenced on a HiSeq X platform, using 1 lane each for the *de novo* and Chicago libraries. This produced **∼**852,000,000 Illumina HiSeq 150 bp read pairs providing **∼**377× physical coverage of the genome (Table 1). The *de novo* genome assembly was constructed with a modified Meraculous assembler. Then we used the Chicago and 20–30X shotgun data to scaffold the newly generated draft assembly with the HiRise^TM^ software pipeline, which produced a draft assembly consisting of 45,357 scaffolds with an N50 of 26.1 Kbp (Table 1). Kmer analysis from raw sequence reads provided an estimated genome size of 0.682 Gbp; Meraculous assembly provided an estimated genome size of 0.673 Gbp (Supplementary Figure S2).

**Table 1:** *Collinsia rattanii* nuclear genome assembly statistics by version

		Illumina HiSeq (Dovetail Genomics)	Hi-C sequencing (Phase Genomics)
Scaffold	Total length (bp)	499,700,000	506,461,782
	Number	45,357	5,029
	N50 length (bp)	26,100	69,250,000
	Longest scaffold (bp)	231,400	95,162,470
Contig	Total length (bp)	475,000,000	506,580,232
	Number	665,422	6,164
	N50 length (bp)	2,000	650,607
	Longest contig (bp)	37,300	NA

To improve on the draft assembly, we sent young *C. rattanii* leaf tissue to Phase Genomics (Seattle, WA, USA) for Hi-C sequencing and genome scaffolding. A Proximo Hi-C library was prepared with chromatin conformation capture data from the new *C. rattanii* tissue sample, following the manufacturer’s protocol. The library was sequenced on an Illumina HiSeq 4000, producing **∼**244,000,000 Illumina HiSeq 100 bp read pairs from Hi-C sequencing providing **∼**72× physical coverage. After aligning these sequences to the draft assembly, putative misjoined contigs were broken with Juicebox according to the observed Hi-C signal, leading to a total of 25 broken contigs (Durand *et al.* 2016). We then re-aligned to the draft assembly using the resulting corrected sequences (excluding duplicates, pairs with low MAPQ or high edit distance, and pairs mapping to contigs <5 Kbp). Finally, we ran Proximo Hi-C scaffolding **∼**200,000 times on the corrected and filtered mappings to optimize scaffold construction and produce our high-quality *C. rattanii* genome (N50 = 69.25 Mbp; Table 1) according to Hi-C signal, as described in Bickhart *et al.* (2017). We recovered orphaned contigs with Juicebox (Durand *et al.* 2016).

We performed gene annotation using data from 6 of the 9 *C. rattanii* RNAseq samples presented and analyzed in this study (see below) plus amino acid sequences from *Erythranthe guttata* (family Phrymaceae, formerly *Mimulus guttatus*) with the BRAKER pipeline (Hoff *et al.* 2019) (Igenbio, Inc., Chicago, IL, USA). *E. guttata* is one of the closest relatives of *Collinsia* with high quality, well-annotated genomic resources (http://phytozome.jgi.doe.gov/pz/p2ortal.html) and used as a model system for understanding floral and mating system evolution (*e.g.*, Martin and Willis 2007; Wu *et al.* 2008). We predicted ribosomal RNAs using barrnap, and tRNAs using tRNAscan-SE [v2.0]. Finally, we assessed the completion of the assembly and gene predictions with BUSCO analysis, using a proprietary Igenbio, Inc., Embryophyta data set (Sim**ã**o *et al.*^2015^).

### Bud development, RNA sequencing, and alignment

Major differences in floral form between *Collinsia* species may be initiated during floral organ development. Therefore, our goal was to compare gene expression patterns between the sister species across floral bud development, after onset of organ development but prior to anthesis. However, the timing of critical events such as differentiation of pollen or ovule precursor cells or maturation of pollen or embryo sacs, and the programming or onset of developmental trajectories including timing of stigmatic receptivity, are not yet known (Kalisz *et al.* 2012). As a first step and to determine repeatable, parallel sampling points, we dissected buds from both species across the continuum of their pre-anthesis development (*C. linearis N* = 34, *C. rattanii N* = 30) and recorded the size and color of the petals, stamens, and style in each. We used these data to divide floral bud development in each species into early, middle, and late pre-anthesis or “bud” stages (B1, B2, B3, respectively) based on distinct color changes in the developing organs and changes in organ size (Table 2; Supplementary Figure S3). We sampled 2–4 whole floral buds per bud stage from each of 3 individuals per species. The three *C. rattanii* individuals originated from two populations in Trinity Co., CA, USA (HFP: 40_**°**_29′19.23″ N, 123_**°**_09′54.50″ W, BC16: 40_**°**_39′22.61″ N, 123_**°**_09′19.82″ W). *C. linearis* individuals originated from two populations in Trinity Co. (MGB: 40_**°**_45′39.6″ N, 123_**°**_39′36.72″ W) and Siskiyou Co. (BARR: 41_**°**_23′12.66″ N, 122_**°**_59′21.42″ W), CA, USA. Plants were germinated at the University of Tennessee-Knoxville, following conditions in Hazzouri *et al.* (2013; Supplementary Table S1) and subsequently shipped to the University of California-Riverside, where they were grown to flowering and buds were collected. We extracted RNA from each bud, using Qiagen RNeasy Plant Mini Kits (QIAGEN, Hilden, Germany) according to the manufacturer’s protocol. RNA quality was verified with a Bioanalyzer (Agilent, Santa Clara, CA, USA). For RNAseq library generation, we used an NEBNext Ultra Directional RNA Library Prep Kit for Illumina and the NEBNext Poly(A) mRNA Magnetic Isolation Module (New England BioLabs, Ipswich, MA, USA), according to the manufacturer’s protocol. We sequenced the libraries on an Illumina HiSeq 2500 platform with 125 bp paired-end reads, at the Genome Innovation Center, McGill University, Montreal, Canada. Raw read quality was verified with FastQC [v0.11.9] (Andrews 2010; Supplementary Table S2).

**Table 2:** *Collinsia* floral bud characteristics by stage

Species	*C. rattanii*			*C. linearis*		
Stage	B1	B2	B3	B1	B2	B3
Bud length (mm)	<2.5	2.5–4.0	>4.0	<2.8	2.8–4.75	>4.75
Petal color	Green	White	Violet	Green	White [violet]^a^	White
Small stamen height (mm)	<1.0	1.0–2.0	>2.0	<1.0	1.0–2.5	>2.5
Large stamen height (mm, linear only)	<1.0	1.0–2.9	>2.9	<1.5	1.5–3.5	>3.5
Large stamen height (mm, curve traced)	<1.0	1.0–2.9	>2.9	<2.0	2.0–3.5	>3.5
Most distinctive anther color	Green	Yellow [brown]	Yellow	Green	Brown	Brown or yellow
Ovary length (mm)	<0.5	0.5–0.9	>0.9	<0.5	0.5–0.9	>0.9
Style length (mm)	<0.5	0.5–2.0	>2.0	<0.5	0.5–1.75	>1.75
Style shape, color	Straight, green	Straight, white [curved]	Curved, purple	Straight, green	Curved, white [straight, green]	Curved, white

^a^ Note: Less common conditions indicated in brackets.

We aligned the raw transcriptome sequences to the *C. rattanii* genome using the STAR 2-pass method [v2.7.3a] (Dobin and Gingeras 2015). RNA-seq reads from each sample that aligned to annotated gene models were summarized in R [v3.6.2] with the “featureCounts” function in the “Rsubread” package [v2.0.1] (Liao *et al.* 2014; R Core Team 2020). We then conducted a regularized logarithm transformation of our raw count data in DESeq2 [v1.26.0] to eliminate the dependence of count variance on count mean; this transformed read count data was used solely for principal components analysis (PCA) and any other gene expression visualizations (Love *et al.* 2014).

### Gene expression analyses

We first explored global patterns of gene expression across the full genome dataset to determine the extent to which genome-wide read count patterns illustrate developmental divergence between sister species and among stages within species. We used PCA with the “FactoMineR” [v2.3] and “factoextra” [v1.0.7.999] R packages (Lê *et al.*^2008^; Kassambara and Mundt 2020) to visualize whether and how our samples clustered based on species and bud stage in relation to the first three PCA axes.

We then identified individual genes showing differential expression between species and developmental bud stages. We modeled a matrix of summarized read counts as the linear combination Y **∼** species + stage + species*stage using the DESeq2 R package [v1.26.0] (Love *et al.* 2014). DESeq2 optimizes the analysis of RNAseq count data from multiple genes by automatically conducting independent filtering (*i.e.*, removing zero-sum or low-count, uninformative genes based on sample count means) and calculating Cook’s distances for outlier detection and removal prior to hypothesis testing (Cook and Weisberg 1982). Because we are primarily interested in evaluating whether changes in gene expression across developmental stages differ in direction or magnitude between the sister species, we focus our results on identifying genes for which the interaction term is significant. Such genes could point toward potential candidates underlying developmental differences associated with the selfing syndrome. We used a likelihood ratio test (LRT) to test for the significance of the interaction term, correcting for multiple tests with a false discovery rate (FDR; Benjamini and Hochberg 1995) of 0.01.

Because DESeq2 treats time as a categorical variable, the LRT for the species*stage interaction functions as an omnibus interaction test, comparing all combinations of stages and species. We, therefore, also used planned contrasts to explicitly compare gene expression changes that occur early (between stages B1 and B2) *vs* late (between stages B2 and B3) in floral bud development between the species. Significance of contrasts was evaluated using a Wald test, which determined whether the estimated log_2_ fold changes in read counts as implicated in each contrast were significantly different from zero (Love *et al.* 2014).

We also identified genes that change in parallel across developmental stages between the species, as these might represent genes controlling floral development that are common to both *C. rattanii* and *C. linearis* (*e.g.*, Sun *et al.* 2016). We first used a LRT to evaluate which genes had a significant “time” (here, “stage”) effect (full model = **∼** species + stage; reduced model = **∼** species), then excluded the subset of the “stage effect” genes that also had a significant “species by stage” interaction effect based on the full model used above.

### Gene ontology enrichment analysis

To functionally profile sets of differentially expressed genes, we performed a gene ontology (GO) enrichment analysis in R package “topGO” [v2.38.1] (Alexa and Rahnenf**ü**hrer 2019). Biological process GO term annotations for each gene were extracted from the ERGO [v2.0] proprietary online database (Igenbio, Inc., Chicago, IL, USA). We used Fisher’s exact test to assess the significance of each GO term and the topGO default (“weight01”) algorithm to account for the hierarchical structure of GO terms in enrichment tests (Alexa *et al.* 2006). We also used REVIGO (http://revigo.irb.hr/) to flag clusters of redundant significant terms (Supek *et al.* 2011).

### Data availability

The following are openly available in figshare: (1) S*upplementary Figures and Tables*, (2) the high quality *C. rattanii* genome assembly, and (3) Supplementary Files (S1–S8), as single zip file, including custom bash/shell and R code used in this project and other supplementary files that allow for replication of the results presented in this study. The raw *C. rattanii* and *C. linearis* RNA-seq read data used in this publication have been deposited in NCBI Gene Expression Omnibus and Sequence Read Archive and are accessible through GEO Series accession number GSE174273 (https://www.ncbi.nlm.nih.gov/geo/query/acc.cgi?acc=GSE174273). Supplementary material is available at figshare: https://doi.org/10.25387/g3.14599995.

## Results

### *Collinsia rattanii* genome assembly and annotation

**Figure 2:**
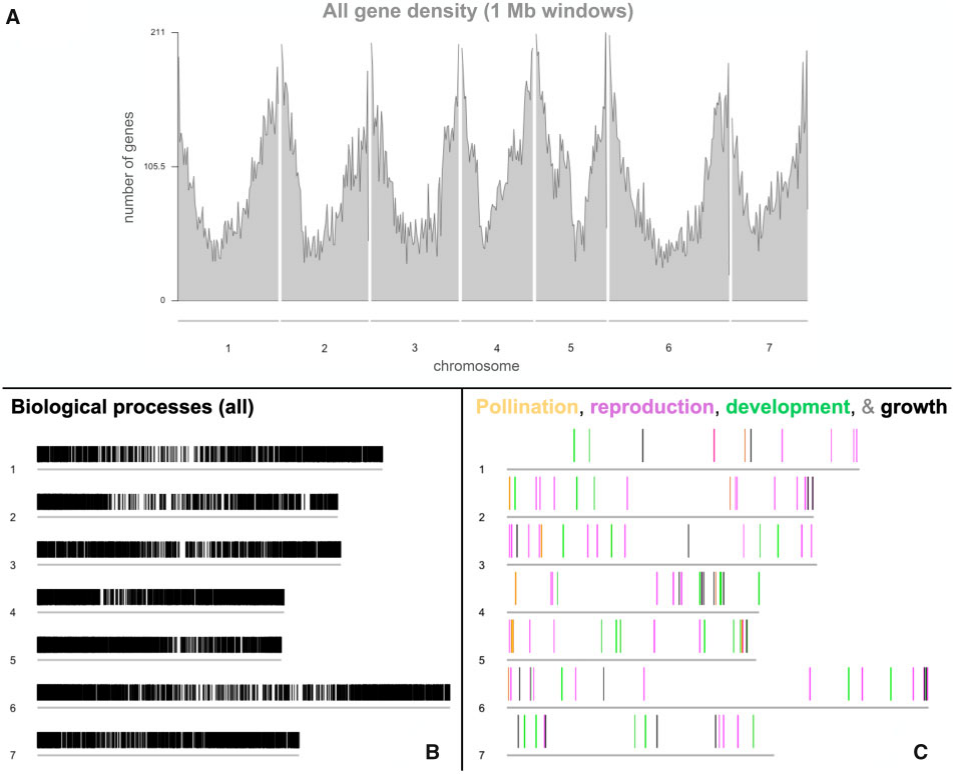
Distribution of *C. rattanii* genes across 7 chromosomes. The 7 major chromosomes are represented in each sub-figure by 7 horizontal gray lines. (A) All gene density with values calculated at 1 Mb windows. The distribution of 45,542 genes is shown; the remaining 1668 genes are located on un-scaffolded contigs. (B, C) Physical maps of functionally annotated *C. rattanii* genes, using GO (gene ontology) terms for biological processes. Chromosome numbers are shown to the left of each chromosome (shown as gray lines). (B) 10,708 genes (*i.e.*, vertical black lines) with one or more GO term annotations for biological processes; another 156 such genes are located on un-scaffolded contigs. (C) Genes annotated with general GO terms for pollination (15 genes in yellow), reproduction (57, in pink), development (31, in green), and growth (20, in black). Some lines may visually overlap one another.

Hi-C contact mapping and scaffolding produced seven major genome scaffolds, as well as 5022 unscaffolded contigs (Lieberman-Aiden *et al.* 2009) (Table 1; Supplementary Figure S4). This high-quality assembly consists of **∼**0.506 Gbp, or **∼**74% of the estimated genome size (Table 1). The ERGO/Igenbio annotation pipeline predicted 47,210 genes (48,523 ORFs) and an average GC content of 35.39% in the *C. rattanii* genome (Table 3; Figure 2A). Gene density per Mb across the genome is shown in Figure 2A. BUSCO analysis recovered 96.3% complete BUSCOs from a total of 1375 BUSCO groups searched, indicating a high level of genome assembly completeness (Simão *et al.* 2015; Table 4). One or more biological process GO term annotations were available for 10,864 genes (**∼**23%; Figure 2, B and C) (Igenbio, Inc., Chicago, IL, USA).

**Table 3:** Gene annotation statistics for *Collinsia rattanii*

Genome features and gene models	Value
Total length (bp)	506,580,232
Contigs (#)	5,029
G + C content (%)	35.39
Total coding bases (#)	94,323,969
Total gene models (#)	47,210
Average coding sequence length (bp)	311
Genes functionally annotated (%)	12.5
tRNA models (#)	1,083
rRNA models (#)	46

**Table 4:** BUSCO scores for *Collinsia rattanii* genome

Metrics	Number (%)
Complete BUSCOs	1,323 (96.3)
Complete and single-copy	1,186 (86.3)
Complete and duplicated	137 (10)
Fragmented BUSCOs	33 (2.4)
Missing BUSCOs	19 (1.3)
Total BUSCO groups searched	1,375 (100)

### Gene expression and gene ontology enrichment

#### Genome-wide expression patterns:

**Figure 3:**
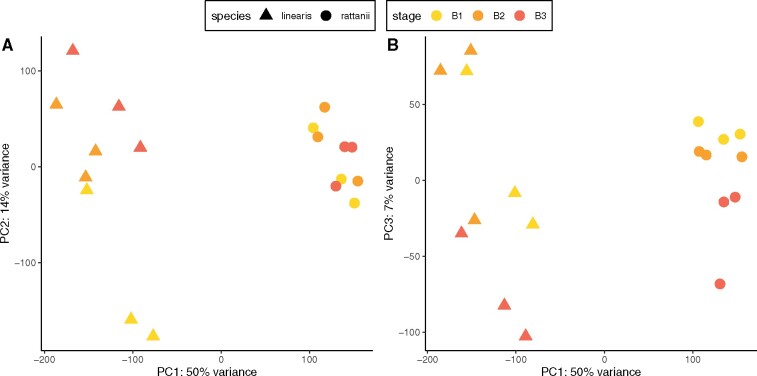
Overall gene expression by species and bud stage. Principal component analysis (PCA) plot of regularized log-transformed RNA-seq read counts for 47,210 (38,589 expressed) genes in the *C. rattanii* genome. *N* = 3 for each species-stage combination. Principal component 1 (PC1) represents 50% of the variance in the read count dataset. (A) PC2 represents 14% of the variance in the read count dataset. Data points are jittered to reduce overlapping. (B) PC3 represents 7% of the variance in the read count dataset.

**Figure 4:**
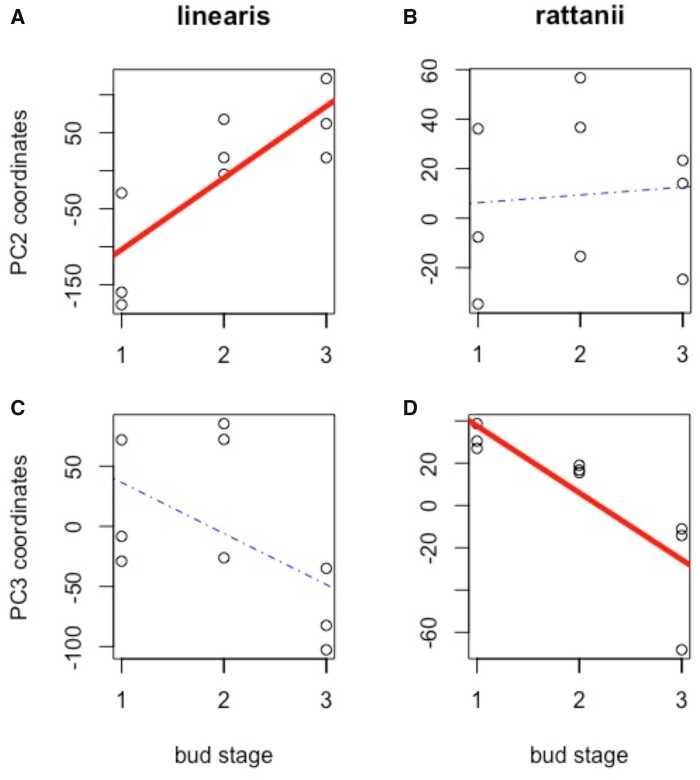
Overall gene expression by species and coordinates. Scatterplots with linear trend lines showing relationships between principal components coordinates and bud stages for *C. rattanii* and *C. linearis.* Each circle represents one sample (replicate). Red, bold trendlines are statistically significant (*P *<* *0.01); dotted trendlines are not significant.

The 18 floral bud samples returned 37.8 million transcripts on average (range 27.2–47.9 million), 80–88% of which, depending on sample, mapped to unique loci in the *C. rattanii* genome (Supplementary Table S2). The percentage of uniquely mapped transcripts did not differ between species (*t*** **=** **0.823, *P*** **=** **0.423; Supplementary Table S2). Of 47,210 total predicted genes, 38,589 (**∼**82%) showed expression by one or more samples and were included in our analyses. PCA on the full gene expression dataset reveals clearly defined species-specific clusters differentiated along the primary axis (PC1), representing 50% of the variance in overall gene expression (Figure 3). Notably, we see greater variation among *C. linearis* samples along this axis. In fact, *C. linearis* samples also show significantly greater variation in expression per gene based on raw read counts of all expressed genes (with a mean read count _**≥**_8) [mean coefficient of variation (CV) = 0.6985] than *C. rattanii* samples (mean CV = 0.5373; *t*** **=** **60.693; *P* < 0.001). The second axis (PC2) represents 14% of the variance in the full dataset and is significantly and strongly correlated with bud developmental stage for *C. linearis* (*r*** **=** **0.82, *P*** **=** **0.007) but not *C. rattanii* (*r*** **=** **0.09, *P*** **=** **0.825) (Figures 3 and 4). Instead, we found that the third axis (PC3), which explains 7% of the variance in the full gene expression dataset, is significantly and highly correlated with bud developmental stage in *C. rattanii* (*r* = -0.83, *P*** **=** **0.006) (Figure 4). Floral bud stage in *C. linearis* shows a weaker, nonsignificant trend with PC3 (*r* = −0.54, *P*** **=** **0.136) (Figure 4). Together, these results indicate that stage of bud development can account for a significant amount of variation in total gene expression in our dataset and reveal greater variation across stages within *C. linearis*. Lower variation across stages in *C. rattanii* could reflect the compression of developmental processes into the shorter time frame of *C. rattanii* flower development, resulting in greater overlap of gene expression across stages.

#### Differential gene expression and gene ontologies:

**Figure 5:**
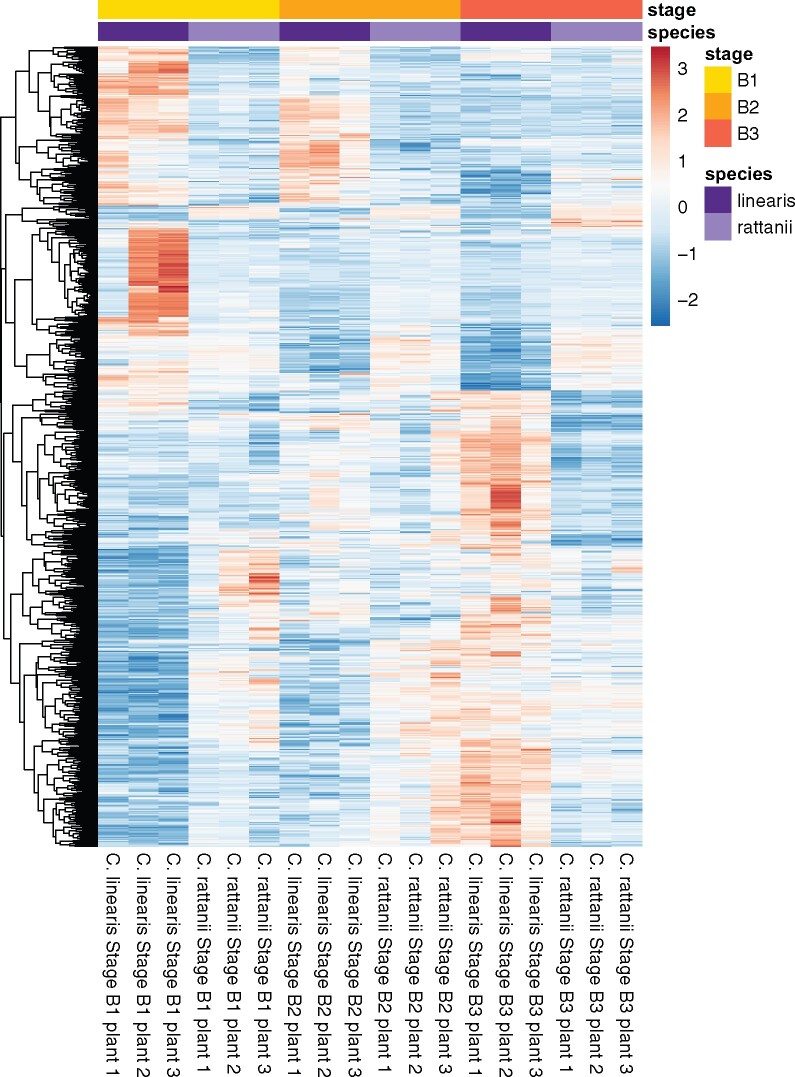
Differential expression of interaction effect genes. Heatmap and hierarchical clustering of regularized log-transformed RNA-seq read counts for the 855 interaction effect genes in the *C. rattanii* genome. Expression levels were further standardized within each row (*i.e.*, within each gene) by subtracting the row mean from each value in a row and dividing those values by the row standard deviation, resulting in a mean of 0 and a standard deviation of 1 for each row. Each row represents one gene’s full expression profile. Red = over expression; blue = under expression. *N* = 3 for each species-stage combination.

We detected a significant “species by stage” interaction effect on the expression of 855 genes (hereafter “interaction effect genes”) based on LRTs (Figure 5) indicating that, for these genes, the direction or magnitude of expression changes between stages is species-dependent. In particular, the differences in expression level, based on read counts, across stages tend to be greater in magnitude in *C. linearis* compared to *C. rattanii* (Figure 5). This is consistent with greater separation of developmental stages across the longer duration of flower development in the outcrosser.

**Figure 6:**
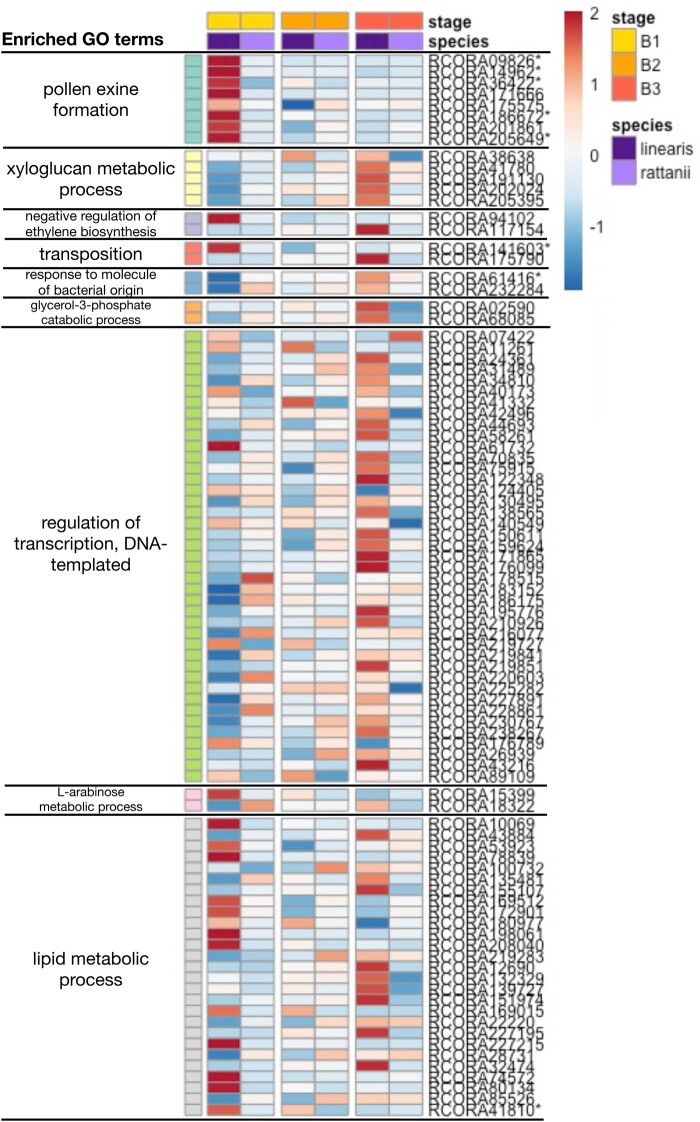
Interaction effect gene expression patterns by biological process. Heatmap of regularized log-transformed RNA-seq read counts (averaged across biological replicates) for the 91-gene subset of interaction effect genes that are also indirectly or directly annotated with the 11 (but see note below) significant GO terms as presented in Table 5. Expression levels were further standardized within each row (*i.e.*, within each gene) by subtracting the row mean from each value in a row and dividing those values by the row standard deviation, resulting in a mean of 0 and a standard deviation of 1 for each row. Each row represents one gene’s full expression profile. Red = overexpression; blue = under expression. *Note: These genes are annotated with 2 different significant GO terms, but only appear here in association with one. Additional annotations include: “sporopollenin biosynthetic pathway” for RCORA09826 and RCORA186672; “lipid metabolic process” for RCORA14962; “callose deposition in cell wall” for RCORA36422 and RCORA205649; and “regulation of transcription, DNA-templated” for RCORA141603, RCORA61416, and RCORA41810.

We could assign **∼**43% of the 855 interaction effect genes to GO terms for biological processes. Enrichment analysis indicates that 11 GO categories are significantly overrepresented in this gene set (Table 5; Supplementary Table S3). We note that there was no evidence of underrepresentation in any GO category in the analyses presented. We visualized expression patterns of the 91 interaction effect genes assigned to these 11 overrepresented GO categories and found consistent differences in patterns of gene expression across bud development between the two species (Figure 6). Most conspicuously, we see that all interaction effect genes associated with pollen exine formation, which encompasses sporopollenin biosynthesis and cell wall callose deposition processes, are highly upregulated in stage B1 in *C. linearis* relative to later stages, but relatively lower and constant across bud development in *C. rattanii* (Figure 6). We also see consistent patterns of expression for interaction effect genes associated with xyloglucan (*i.e.*, cell wall hemicellulose) metabolism, glycerol-3-phosphate catabolism, and transcription regulation. For these genes, expression increases steadily across the three stages for *C. linearis*, whereas relatively little change is seen across bud development in *C. rattanii* (Figure 6). Genes associated with lipid metabolism also reflect greater changes in gene expression during bud development in the outcrosser; in this case, about half of the genes are upregulated in B1 relative to other stages in *C. linearis*, similar to the pattern of expression change in pollen exine formation genes, whereas the other half mimic the pattern seen for xyloglucan metabolism, glycerol-3-phosphate catabolism, and transcription regulation. Overall, these results reveal greater changes in gene regulation across bud development in *C. linearis* compared to *C. rattanii*.

**Table 5:** Biological processes of interaction effect genes

	GO ID	Description	Total count of genes in *C. rattanii* genome	Observed count of genes in set	Expected count of genes in set	*P*-value^a^
1	GO:0010584	Pollen exine formation	16	8	0.54	2.00E−06
2	GO:0010411	Xyloglucan metabolic process	30	5	1.02	0.0031
3	GO:0010366	Negative regulation of ethylene biosynthesis	3	2	0.1	0.0034
4	GO:0032196	Transposition	3	2	0.1	0.0034
5	GO:0080110	Sporopollenin biosynthetic process	3	2	0.1	0.0034
6	GO:0002237	Response to molecule of bacterial origin	3	2	0.1	0.0034
7	GO:0046168	Glycerol-3-phosphate catabolic process	3	2	0.1	0.0034
8	GO:0006355	Regulation of transcription, DNA-templated	911	44	30.87	0.0034
9	GO:0046373	L-arabinose metabolic process	4	2	0.14	0.0066
10	GO:0052543	Callose deposition in cell wall	4	2	0.14	0.0066
11	GO:0006629	Lipid metabolic process	602	28	20.4	0.0084

^a^ Significance (*P *<* *0.01) determined via the Fisher statistic.

**Figure 7:**
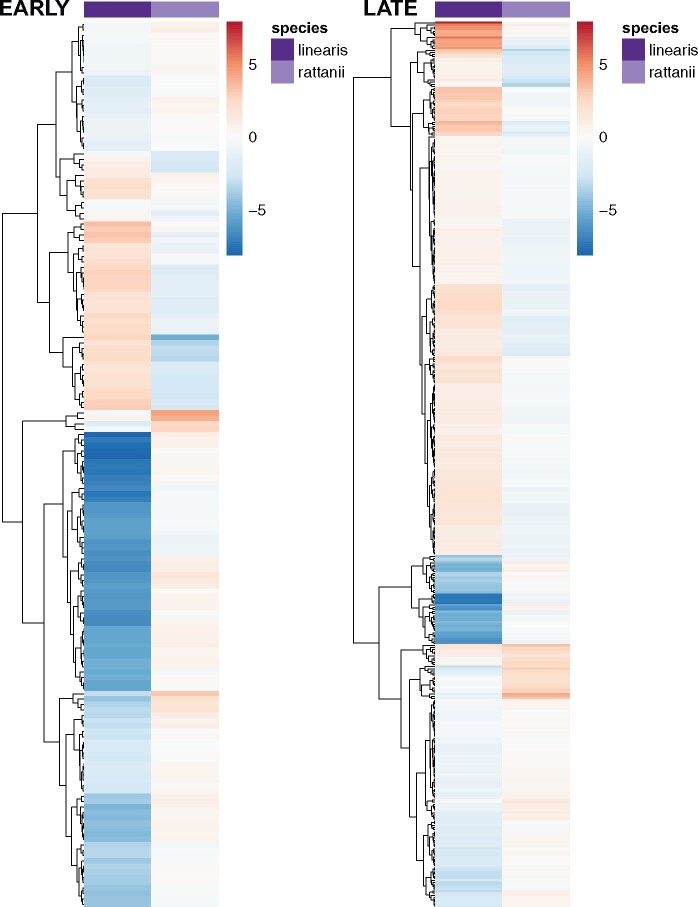
Magnitude and direction of expression changes in early and late development. Heatmaps representing magnitude and direction of significant expression change between bud stages in 164 early and 418 late development contrast effect genes for each species and hierarchical clustering of regularized log-transformed RNA-seq read counts for each gene. Red = positive expression change; blue = negative expression change.

We then focused our examination on changes in expression between sequential stages of bud development corresponding to early (between stages B1 and B2) *vs* later (between stages B2 and B3) development. Based on our planned contrasts, we identified 569 genes for which expression changes in early and/or later development differ in either direction or degree between the species (hereafter “contrast effect genes”; Figure 7), 117 of which were not detected by the LRT. In nearly all cases (77%), the contrast was significant because of up- or down-regulation of the gene in one species but no expression change in the other; in the remaining cases, both species showed changes in expression, but in opposite directions. We also note that whereas the overwhelming majority of these genes show significant differences in regulation between the species in late development (418/569), differences in early development are greater in magnitude (Figure 7).

**Figure 8:**
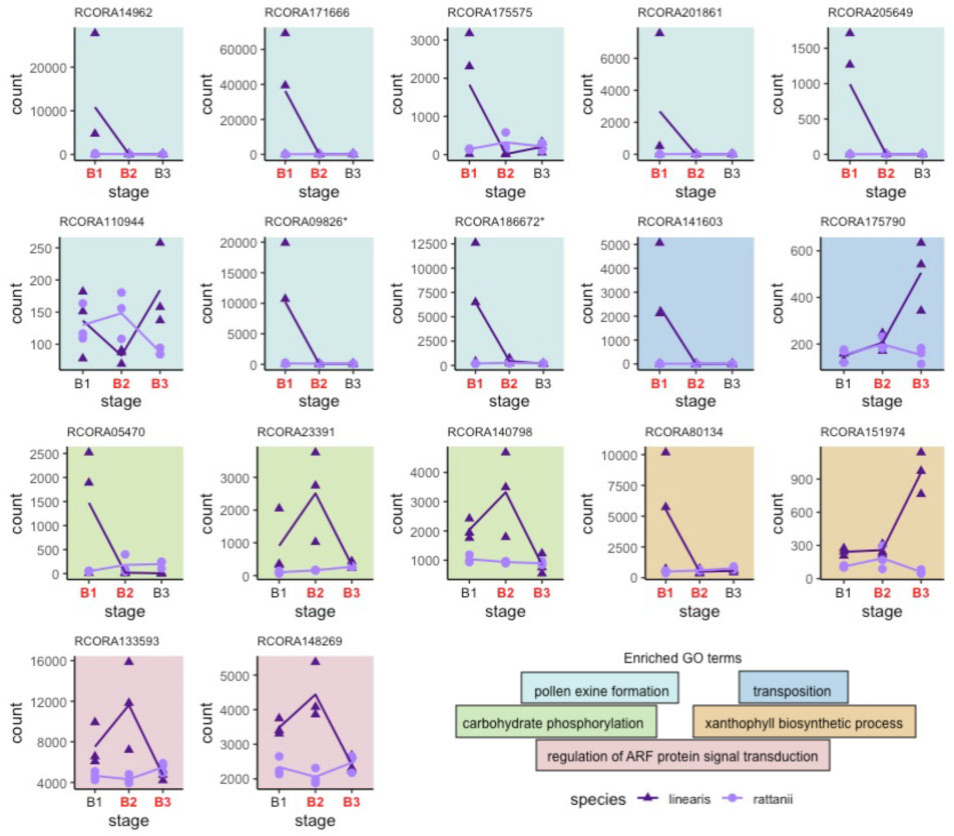
Contrast effect gene expression patterns by biological process. Expression profiles for 17 genes showing significantly different patterns between the species in either early or late development and belonging to the 6 enriched GO categories corresponding to Table 6. RNA-seq read counts (*y*-axis) for each replicate are presented as either triangles (*C. linearis)* or circles (*C. rattanii*); lines connect species-specific count means. On the *x*-axis, stages in red (bold) indicate whether significant differences between species in gene expression profiles occur early (B1–B2) or late (B2–B3) in development. Note: Since GO term “sporopollenin biosynthetic process” is a subprocess of “pollen exine formation,” we categorize gene profiles according to 5 GO terms. *These two genes are also annotated by the significantly enriched GO term “sporopollenin biosynthetic process.”

Based on GO assignments available for 231 of 569 contrast effect genes, we found significant enrichment for six GO terms (Table 6; Supplementary Table S4) and visualized expression patterns of the associated genes (Figure 8). The three GO categories with the greatest over-representation are pollen exine formation, transposition, and sporopollenin biosynthetic process, which are also overrepresented in the larger set of interaction effect genes. Paralleling the overall set of contrast genes, we see the dominant pattern is strong downregulation of expression across early development for *C. linearis* but consistently low expression in *C. rattanii*. Also for *C. linearis*, four out of five contrast effect genes with functions related to ARF protein signal transduction and carbohydrate phosphorylation have large peaks of expression at stage B2, a pattern which is absent (or even inverted) for *C. rattanii* (Figure 8). In general, we see lower magnitudes of changes in gene expression between developmental stages in *C. rattanii* relative to *C. linearis* for contrast effect genes in these enriched GO categories (Figure 8). Therefore, overall reduced temporal fluctuations in gene expression in the selfer relative to the outcrosser is corroborated across both our genome-wide (Figure 3) and various differential expression analyses, which examine disparate gene sets (Supplementary Figure S5).

**Table 6:** Biological processes of contrast effect genes

	GO ID	Description	Total count of genes in *C. rattanii* genome	Observed count of genes in set	Expected count of genes in set	*P*-value^a^	Number of observed DEGs in ED, LD^b^
1	GO:0010584	Pollen exine formation	16	8	0.34	1.30E−07	7, 1
2	GO:0032196	Transposition	3	2	0.06	0.0013	1, 1
3	GO:0080110	Sporopollenin biosynthetic process	3	2	0.06	0.0013	2, 0
4	GO:0016123	Xanthophyll biosynthetic process	6	2	0.13	0.0064	1, 1
5	GO:0032012	Regulation of ARF protein signal transduction	6	2	0.13	0.0064	0, 2
6	GO:0046835	Carbohydrate phosphorylation	20	3	0.43	0.0083	1, 2

^a^ Significance (*P *<* *0.01) determined via the Fisher statistic.

^b^ Number of genes associated with each GO term (Figure 8) showing significant changes in expression during early floral development (ED) and late floral development (LD).

Finally, we found 1022 genes changing in parallel across bud development in the two species (hereafter “shared pattern DEGs”), which could point toward genes underlying processes and mechanisms common to both species and that occur similarly in *C. rattanii, C. linearis*, and, potentially, their close relatives. Based on GO assignments available for 415 of 1022 genes, this set of genes is significantly enriched (*P*** **<** **0.01) for 15 GO terms, and over half of these terms point to plant hormone response, intra- and extra-cellular localization and transport, and/or reproductive developmental processes (Supplementary Table S5).

## Discussion

The genus *Collinsia* is an emerging model system for studying evolutionary genomic mechanisms underlying the shift to selfing and the selfing syndrome. However, until now, progress was hindered by the lack of necessary genomic resources. We report a high-quality *de novo* genome sequence for the highly selfing species *C. rattanii*, with contiguity across its 7 chromosomes (Garber 1958). This sequence information will enable future large-scale genomic studies into the proximate and ultimate causes of mating system evolution within *Collinsia*, which exhibits multiple transitions from predominantly outcrossing or mixed mating to highly selfing. Because high rates of selfing in *Collinsia* do not involve the loss of self-incompatibility found in most model systems of mating system evolution, genomic studies in *Collinsia* have the potential to provide novel insight into the role of developmental trait changes and their molecular basis in the selfing syndrome.

Our gene expression analyses represent a first step toward uncovering molecular mechanisms differentiating floral development in selfing *C. rattanii* and its outcrossing sister *C. linearis*. We reveal less variation in overall gene expression among individuals and bud stages in *C. rattanii* relative to *C. linearis*. These results are consistent with greater heterozygosity of outcrossing *C. linearis* individuals compared to highly selfing, homozygous *C. rattanii* demonstrated previously by Hazzouri *et al.* (2013) and greater phenotypic and genetic variation seen across outcrossing plant species more broadly (Hamrick and Godt 1996; Kalisz *et al.* 2012). Moreover, we find that gene expression was generally lower and changed relatively little across bud development in *C. rattanii* compared to *C. linearis*. Our findings suggest that transcriptional regulation of enzymes involved most notably in pollen exine formation, including sporopollenin biosynthesis, may influence floral traits that distinguish selfing and outcrossing *Collinsia* species through pleiotropic functions.

The strongest pattern that arose from our dataset is the substantially greater expression of multiple genes associated with pollen exine formation in stage B1 in *C. linearis* relative to *C. rattanii.* This finding aligns with previous gene expression analysis in *C. rattanii* and *C. linearis* indicating generally lower expression of genes important in pollen production, flavonoid production, and morphogenesis in *C. rattanii* (Table 4, Supplementary Table S2 in Hazzouri *et al.* 2013). Here, we isolate this difference to early stages of floral bud development. This timing may reflect the fact that the anthers are well developed early in bud development relative to other floral organs (Supplementary Figure S3). Typical of the difference between selfing and outcrossing species, the outcrosser *C. linearis* produces more pollen than *C. rattanii*. Thus, the difference in expression profiles of pollen-related genes seen in our study may be associated with differences in the number of cells in tissues dedicated to pollen production and cellular construction in each species. Reduction in the number of pollen grains per flower is commonly seen in selfing species (Cruden 1977; Goodwillie *et al.* 2010; Tsuchimatsu *et al.* 2020). High pollen production is likely under directional selection and adaptive for plants that rely primarily on outcrossing as it increases siring opportunities, but the evolution of the selfing syndrome may have either relaxed this selective pressure for *C. rattanii* or reversed its direction, favoring limited pollen production in the face of resource allocation trade-offs. Evidence for the latter was recently found in the selfing plant *Arabidopsis thaliana* (Tsuchimatsu *et al.* 2020). Of note, several differentially expressed genes between our focal *Collinsia* species are orthologous to those in *A. thaliana.* A gene encoding a protein associated with pollen exine formation and sporopollenin biosynthesis (*C. rattanii* genome designation *RCORA09826*), which shows substantially greater expression in stage B1 in *C. linearis*, is orthologous to *A. thaliana* gene *AT4G34850*, a chalcone synthase (ERGO/Igenbio, Inc. 2020), and to *LESS ADHESIVE POLLEN5 (LAP5)*. Another gene that similarly shows greater expression in stage B1 in *C. linearis* (*RCORA03278*) is also orthologous to *A. thaliana LESS ADHESIVE POLLEN6 (LAP6)*. The *LAP* genes encode chalcone synthase-like proteins for pollen exine formation in anthers (Dobritsa *et al.* 2010; ERGO/Igenbio, Inc. 2020). Chalcone synthase in plants is also needed for flavonoid production and thus pollinator-attracting pigments in petals as well as functional pollen production and pollen-stigma adhesion (Mo *et al.* 1992; Dobritsa *et al.* 2010). Mutants with reduced chalcone synthase expression have been produced in several species, resulting in production of white, sterile anthers (*Petunia* spp.; Napoli *et al.* 1999), white corollas (*Ipomoea* spp.; Durbin *et al.* 1995), and smaller fruits with reduced flavonoid concentrations (*Malus* ×*domestica*; Dare *et al.* 2013). Therefore, low relative levels of genes *RCORA09826* and *RCORA03278* expression in *C. rattanii* throughout floral bud development are consistent with observed selfing syndrome morphological trends (Figures 6 and 8).

We also saw a trend of significantly increased expression of genes associated with xyloglucan metabolism, glycerol-3-phosphate catabolism, regulation of transcription, and lipid metabolism in late bud development in *C. linearis* (Figures 6, Supplementary Figure S6). Xyloglucan is a cell wall carbohydrate, the modification of which is associated with plant growth, including that of floral organs (*e.g.*, stamens, petals; Kurasawa *et al.* 2008; Harada *et al.* 2011). The increased expression of these genes in *C. linearis* in the latest bud stage could be related to the larger flowers that are characteristic of *C. linearis* relative to *C. rattanii* and thus greater growth seen during stage B3 (Supplementary Figure S3). Likewise, both DEGs associated with glycerol-3-phosphate catabolism putatively encode glycerol-3-phosphate dehydrogenase, a highly conserved enzyme critical to cell membrane synthesis and glycolysis (Yeh *et al.* 2008). Regarding lipid metabolism genes, increased expression may reflect pollen maturation occurring closer to anthesis combined with the greater number of pollen grains in *C. linearis* (Zhang *et al.* 2016).

Ultimately, our work provides new genomic resources for a developing model of mating system evolution and first steps to understanding the role of gene expression. Future work will extend broadly to additional *Collinsia* outcrossing-selfing species pairs to identify signatures of parallel molecular and genomic evolution as well as go deeper to identify eQTL associated with contrasting mating-system traits in *C. ratannii* and *C. linearis*. Moreover, there is an abundance of existing ecological and phenotypic data on *Collinsia* species related to niche breadth (Randle *et al.* 2009; Grant and Kalisz 2020), sexual interference, sexual conflict, and pollen traits (*e.g.*, Lankinen and Madjidian 2011; Lankinen *et al.* 2016; Lankinen and Strandh 2019; Malagon *et al.* 2019; Madjidian *et al.* 2020), pollination biology (*e.g.*, Elle and Carney 2003; Kalisz and Vogler 2003; Kalisz *et al.* 2004; Randle *et al.* 2018), phenotypic plasticity (Jorgensen and Arathi 2013; Spigler and Kalisz 2013), population differentiation (Hazzouri *et al*. 2013; Salcedo *et al*. 2014) and adaptation to extreme soil types (Wright *et al.* 2006; Moyle *et al.* 2012). Consequently, the development of genomic resources in *Collinsia* has potential to pave the way for a broad array of ecological genomic studies.
